# Supplementary material for: Age-Related Associations of Altruism with Attitudes towards COVID-19 and Vaccination: A Representative Survey in the North of Italy
Source: Behav Sci (Basel). 2023 Feb 19;13(2):188. doi: 10.3390/bs13020188 (PMC9951972; doi:10.3390/bs13020188)
Supplement: Supplementary file 1 [file behavsci-13-00188-s001.zip › behavsci-2176864-supplementary.pdf]

behavioral sciences

MDPI

Supporting Material

Age-Related Associations of Altruism with Attitudes toward COVID-19 and Vaccination: A Representative Survey in the North of Italy

Verena Barbieri, Christian J. Wiedermann, Stefano Lombardo, Barbara Plagg, Giuliano Piccoliori, Timon Gärtner, and Adolf Engl

Table S1. Medians and 1st and 3rd quartiles of altruism sums scores for demographic characteristics of the sample by age groups

Variable	Altruism sum score									
	Overall			Age 18 – 69 years			Age 70 years or more			
	**N (%)**	**Median [1Q;3Q]**	***p*-value†**	**N (%)**	**Median [1Q;3Q]**	***p*-value†**	**N (%)**	**Median [1Q;3Q]**	***p*-value†**	***p*-value***
Total	1,426 (100)	24 [20;26]	---	1,169 (100)	23 [20;26]	---	257 (100)	24 [20;27]	---	< 0.05
Gender			< 0.001			< 0.001			<0.05	
Male	692 (48.5)	22 [19;25]		581 (49.7)	22 [19;25]		110 (42.8)	23 [19.1;26]		n.s.
Female	734 (51.5)	25 [21;27]		588 (50.3)	25 [21;27]		147 (57.2)	25 [21;28]		n.s.
Education			n.s.			n.s.			n.s.	
Middle school or lower	316 (22.2)	24 [20;27]		182 (15.6)	24 [20;27]		134 (52.3)	24 [19;27]		n.s.
Vocational school	411 (28.8)	23 [20;26]		348 (29.8)	23 [19;26]		63 (24.6)	25 [20;28.5]		< 0.05
High school	411 (28.8)	23.5 [20;26]		371 (31.8)	23 [20;26]		39 (15.2)	25.5 [22;28.5]		< 0.01
University	288 (20.1)	24 [21;26.5]		267 (22.9)	24 [21;26]		20 (7.8)	23 [21;26.3]		n.s.
Citizenship			<0.01			<0.01			n.s.	
Italian	1308 (91.7)	24 [20;26]		1058 (91.6)	23 [20;26]		249 (97)	24 [20;27]		< 0.01
Other	118 (8.3)	25 [20;27.5]		110 (9.4)	25 [20;27]		8 (3.0)	27.2 [21;30]		n.s.
Native Language ‡			n.s.			<0.05			n.s.	
German	879 (61.7)	24 [20;27]		722 (61.8)	2 [20;26]		156 (61.0)	24 [20;27.2]]		< 0.01
Italian	384 (26.9)	23 [20;26]		294 (25.2)	24 [20;27]		90 (34.9)	24 [20;27]		n.s.
Ladin	57 (4.0)	23 [20;28.4]		48 (4.1)	23 [20.6;27.1]		9 (3.5)	27 [20;30]		n.s.

Other/more than one	106 (7.4)	24.3 [20;27]		104 (8.9)	25 [20;27]		1 (0.6)	22 [22;22]		n.s.
Household / Family structure (more than one answer possible)										
Single	236 (16.6)	24 [20;27.5]	0.012	151 (12.9)	23.3 [20;28]	n.s.	85 (33.1)	24.4 [20;27.2]	n.s.	n.s.
Children 0-17 years of age	405 (28.4)	24 [20;27]	n.s.	400 (34.2)	24 [26;26]	n.s.	5 (1.8)	27 [12;29]	n.s.	n.s.
COVID-19 patient at risk[2]	302 (21.2)	24 [21;27]	0.047	186 (15.9)	24 [21;27]	n.s.	115 (44.9)	25 [21;27]	n.s.	n.s.
None of the above	522 (36.7)	2 [20;26]	0.030	469 (40.1)	23 [20,26]	n.s.	53 (20.8)	22 [19;26]	< 0.05	n.s.
Working in the health sector			< 0.001			< 0.001			n.s.	
Yes	85 (5.9)	25 [22;27]		83 (7.1)	25 [22;27]		2 (0.6)	27 [n.a.]		n.s.
No	1,341 (94.1)	23 [20;26]		1086 (92.9)	23 [20;26]		255 (99.4)	24 [20;27]		<0.01
Chronic disease(s)			< 0.001			0.010			< 0.05	
Yes	246 (17.3)	25 [21;28]		141 (12.0)	24 [21;28]		106 (41.2)	25 [20;28]		n.s.
No	1,179 (82.7)	23 [20;26]		1028 (88.0)	23 [20;26]		151 (58.8)	24 [20;27]		n.s.
COVID-19 infected			n.s.			n.s.			n.s.	
Yes	252 (17.7)	23 [26;26]		218 (18.7)	23 [20;26]		33 (13.0)	20 [15.9;25.1]		< 0.05
No	1,174 (82.3)	24 [20;27]		950 (81.3)	24 [20;26]		223 (87.0)	25 [21;27]		< 0.01
Economic situation (last 3 months)			n.s.			n.s.			n.a.	

Better	43 (3.0)	24 [20;26]		42 (3.6)	24 [19.5;26]		1 (0.6)	27 [n.a.]		n.s.
The same	973 (68.2)	24 [20;27]		751 (64.3)	24 [20;26]		222 (86.4)	25 [20;27]		< 0.05
Worse	375 (26.3)	23 [20;26]		346 (29.6)	23 [20;26.8]		29 (11.3)	23 [22;25]		n.s.
Don`t know	35 (2.4)	21.6 [17.4;25.2]		30 (2.6)	22 [17.7;24.8]		5 (1.8)	18 [13;30]		n.s.
Urban/rural residency			0.046			0.047			n.s.	
Urban	602 (42.2)	24 [20;27]		487 (41.7)	24 [20;27]		114 (44.5)	25 [20;27]		n.s.
Rural	824 (57.8)	23 [20;26]		681 (58.3)	23 [20;26]		142 (55.5)	24 [20;27]		< 0.05
Vaccine hesitancy			n.s.			n.s.			n.s.	
Hesitant	222 (15.6)	23 [20;27]		208 (17.8)	24 [20;26]		14 (5.3)	23 [20.6;27]		n.s.
Non-hesitant	1,204 (84.4)	24 [20;26]		961 (82.2)	23 [19;27]		243 (94.7)	24 [20;27]		< 0.05

† *p*-values refer to Man Whitney-test and Kruskal-Wallis-Test, respectively to baseline characteristic groups for altruism.
* *p*-values refer to Chi-Square-Test and compare baseline characteristics for differences between age groups.
‡ Participants living together with a COVID-19 patient at risk in one household.
n.a., not available.

Table S2. Spearman correlation coefficients between altruism and attributes of the sample towards opinions about SARS-CoV-2 and SARS-CoV-2 vaccination

Survey item	Correlations with altruism†		
	Total	18–69 years	70 years or more
**Do you agree with the decisions taken by the authorities?**			
Do you agree with the decisions taken by the authorities to increase the coverage of COVID-19 vaccinations?	0.160 ***	0.119 ***	0.269 *
Do you agree with the decisions taken by the authorities to increase the coverage of compulsory vaccinations?	0.161 ***	0.103 **	0.258 ***
Do you agree with the decisions taken by the authorities regarding COVID-19?	0.148 ***	0.125 ***	0.261 **
**Do you support the following actual restrictions regarding COVID-19 ?**			
Mandatory face masks in closed public rooms	0.188 ***	0.212 ***	n.s.
Mandatory face masks in the open air	0.134 ***	0.138 ***	n.s.
Distance learning in parts for scholars from 14 to 19	n.s.	n.s.	n.s.
Distance learning in parts for scholars from 6 to 14	n.s.	n.s.	n.s.
Restricted opening hours for bars and restaurants	0.112 ***	0.105 **	0.125 *
Distance rules in closed public rooms	0.183 ***	0.197 ***	n.s.
Distance rules in the open air	0.149 ***	0.164 ***	n.s.
Restrictions on not individual phisical and sports activities	0.057 *	n.s.	n.s.
Closure of national borders	0.071 *	n.s.	0.124 *
Closure of regional borders	0.119 ***	0.113 ***	n.s.
Compulsory self-isolation after contact with infected persons	0.218 ***	0.228 ***	0.126 *
As much smart working as possible	0.090 **	0.086 **	n.s.
Lockdown	0.069 *	n.s.	0.127 *
Total distance learning	n.s.	n.s.	n.s.
Prohibition of visiting friends and parents not living in the same household	0.089 **	0.079 *	n.s.
Closure of comunal borders	n.s.	n.s.	n.s.
Closure of hotels	0.091 **	0.073 *	n.s.
Limitation of individual phisical or sportive activities	-0.092 **	-0.112 ***	n.s.
**Did you take the following measure within the last 7 day to avoid a COVID-19 infection?**			
Washing my hands often with water and soap for 20 seconds	0.255 ***	0.259 ***	0.224 ***
Avoiding touching your eyes, nose, and mouth with unwashed hands	0.211 ***	0.187 ***	0.291 ***
Use of disinfectants to clean hands when soap and water is not available for washing hands	0.205 ***	0.178 ***	0.316 ***
To renounce social events	0.165 ***	0.151 ***	0.201 **
Using antibiotics to prevent or treat the Corona virus	n.s.	n.s.	n.s.
Wearing a face mask in public	0.235 ***	0.229 ***	0.210 **

Social distancing in the public	0.204 ***	0.168 ***	0.319 ***
Disinfect superficies	0.169 ***	0.150 ***	0.241 ***
Avoid dirct contact with other people	0.112 **	0.089 *	0.158 *
Open the window	0.183 ***	0.185 ***	0.158 *
**What is your position on the anti-COVID-19 vaccine?**			
I believe the vaccine can help contain the spread of COVID-19	0.063*	n.s.	0.165**
If I knew I was already infected with COVID-19, I would not get the vaccine	-0.088 **	-0.082 **	n.s.
If everyone else is vaccinated against COVID-19, then I should not get vaccinated	-0.146 ***	-0.141 ***	-0.143 *
If the vaccine was recommended for me, I would do it	0.105 ***	0.089 **	0.142 *
If your doctor recommended a COVID-19 vaccination, how likely would you be to get vaccinated?	0.090 **	0.065 *	0.182 **
**Which of the following statements on COVID19 vaccination do you agree with?**			
**…the COVID-19 vaccination is not necessesary, because…**			
…it is not effective	-0.072 **	n.s.	-0.183 **
…natural herd immunity is achieved with the spread of the virus and that's enough	-0.100 ***	-0.069 *	-0.202 **
…this disease does not exist/ is a normal flu	-0.108 ***	-0.125 ***	n.s.
…the whole is just a profit for the pharmaceutical industry	-0.108 ***	-0.087 **	-0.173**
**…the covid-19 vaccination is harmful, because,…**			
…long-term risks are unknown	-0.062 *	-0.073 *	n.s.
…new vaccines carry additional risks in the RNA	-0.111 ***	-0.099 **	-0.113 *
…there are doctors who advise against it	-0.077 **	n.s.	-0.114 *
…an obligation to vaccinate certain groups with priority will lead to great socio-political discussions	n.s.	n.s.	n.s.

† Spearman's rank correlation coefficient: altruism sum score against sample attribute defined on a 6-point Likert-scale responses. *p*–values < 0.001 are indicated with ***, < 0.01 with **, < 0.05, *, and *p*–values ≥ 0.05, are regarded as not significant (n.s.)-
